# The effectiveness and safety of acupoint catgut embedding for the treatment of primary dysmenorrhea: A protocol for systematic review and meta-analysis

**DOI:** 10.1097/MD.0000000000023222

**Published:** 2020-11-20

**Authors:** Xue Wang, Nan Wang, Junquan Liang, Yunxiang Xu, Guizhen Chen

**Affiliations:** a Clinical Medical College of Acupuncture, Moxibustion and Rehabilitation, Guangzhou University of Chinese Medicine, Guangzhou; b The Bao‘an District TCM Hospital, The Affiliated Hospital of Guangzhou University of Chinese Medicine, Shenzhen, China.

**Keywords:** acupoint catgut embedding, meta-analysis, primary dysmenorrhea, protocol, systematic review

## Abstract

**Background:**

Primary dysmenorrhea (PD), also regarded as functional dysmenorrhea, refers to dysmenorrhea without obvious organic lesions in the reproductive system. It accounts for more than 90% of dysmenorrhea and seriously affects womens life and work. Previous studies have proved that acupoint catgut embedding therapy is effective and safe for PD patients. It could relieve the pain rapidly and work for a long time in multiple mechanisms. This protocol aims to evaluate the effectiveness and safety of acupoint catgut embedding therapy on PD systematically. With the latest published evidence, a systematic review and meta-analysis of catgut embedding for patients with PD would be carried out in this study.

**Methods:**

All randomized controlled trials (RCTs) related to acupoint catgut embedding therapy on PD will be searched in the following electronic databases: PubMed, Cochrane Library, EMBASE, Wed of Science, Chinese National Knowledge Infrastructure (CNKI), Chongqing VIP Database, Wanfang Database, and Chinese Biomedical Literatures Database (CBM), from inception to September 2020. The primary outcomes contain visual analog scale (VAS), dysmenorrhea symptom score, and clinical effectiveness rate, while the secondary outcomes consist of adverse events and the recurrence rate. Two reviewers will independently perform data selection, data synthesis, and quality assessment. Assessment of risk of bias and data synthesis would be performed with Review Manager 5.3 software.

**Result:**

This systematic review will summarize the current and high-quality evidence of acupoint catgut embedding therapy on PD.

**Conclusion:**

This systematic review aims to offer the latest persuasive evidence for clinical practitioners that using acupoint catgut embedding therapy on PD is effective and safe.

**PROSPERO registration number:**

CRD42020156362.

## 1 Introduction

Primary dysmenorrhea (PD), characterized by abdominal pain along with spasmodic and bloating feelings before or during menstruation, is a very prevalent gynecological disease.^[1]^ It often occurs within 0.5 to 2 years after menarche in young women aged between 15 and 25. In severe cases, patients even suffer from fainting and shock. Compared with secondary dysmenorrhea, PD is defined as the absence of organic pathologic changes in the reproductive system.^[2]^ It is reported that the prevalence of dysmenorrhea varies from 50% to 90% all around the world.^[3]^ According to the epidemiological survey, the incidence of dysmenorrhea among Chinese women is 33.1%. 53.2% of these patients are suffering from PD, and 13.5% are seriously affected in mind and body.^[4]^ PD has been one of the leading causes of work and study absenteeism among young women.^[5,6]^ Fortunately, there are many clinical treatments for PD. In modern medicine, compound norethindrone, compound megestrol, progesterone, ibuprofen, acetaminophen, other hormones, antipyretic and analgesic drugs, and non-steroidal anti-inflammatory drugs are clinically commonly used.^[2]^ These medicines are effective immediately but inferior in the long term. Moreover, patients could have considerable side effects, such as nausea, vomiting, dizziness, headache, and other adverse reactions with long-term use of such drugs.^[7]^

Traditional Chinese medicine (TCM) has treated PD over 2000 years. In TCM, the pathogenesis of PD is summarized in 2 aspects: insufficiency or obstructed qi and blood of the thoroughfare vessel and conception vessel.^[8,9]^ Thus, recuperating qi and blood of the thoroughfare vessel and conception vessel is taken as the critical point to treat PD. There are multiple featured therapies such as Chinese herbal medicine, acupuncture, moxibustion, massage, qigong, etc. Previous studies have shown that acupuncture has a great effect on treating patients with PD. It could regulate qi and blood to relieve the pain. In recent years, several meta-analyses related to common acupuncture, warm acupuncture or electro acupuncture on PD have been published in various journals.^[10]^

Acupoint catgut embedding develops on the combination of traditional acupuncture and modern technology. With the theoretical guidance of “keeping it for a long time to cure chronic diseases”, this therapy takes use of the stimulation from the physicochemical change of the suture in vivo for acupoints.^[11]^ Compared to other therapies, acupoint catgut embedding has the advantages of fewer side effects, faster effect, and higher patient compliance.^[12]^

In recent years, an increasing number of clinical research on acupoint catgut embedding for PD has been carried out. Most of them lack large samples because the long time interval between visits in this therapy may cause the measurement error of observation indexes in large-scale randomized clinical trials (RCTs) easily.^[13]^ So far there is no systemic review of acupoint catgut embedding for the treatment of PD. And the latest meta-analysis of it was published in Chinese 4 years ago. Bringing 9 trials into the study, Wu et al indicated that acupoint catgut embedding is safe for PD patients and could relieve menstrual pain in the long term.^[14]^ But the number of included trials and the sample size of them were both small, and the methodological quality was generally low. These limitations influenced the quality of the meta-analysis. In the past 4 years, several new relevant RCTs which should be incorporated into the analysis appeared. Therefore, we consider it is necessary to conduct the systematic review and update the meta-analysis to access the effectiveness and safety of acupoint catgut embedding on PD and provide strong evidence for the clinic.

## 2 Methods

### 2.1 Registration

This study has been registered at PROSPERO. The review would be carried out in accordance with the preferred reporting items for systematic reviews and meta-analyses (PRISMA) statement guidelines and follow the advice of the Cochrane Handbook for Systematic Reviews of Interventions.^[15,16]^

### 2.2 Type of studies

To evaluate the effectiveness and safety of acupoint catgut embedding for patients with PD, we will include all RCTs published in Chinese or English which explored the specific effect of acupoint catgut embedding in the treatment of PD. Animal experiments, reviews, case reports, and studies with repeated publication or incomplete data will be excluded.

### 2.3 Types of participants

Participants in those included RCTs must be diagnosed with PD, which is defined by the Clinical Guideline of PDs diagnostic standards from the Society of Obstetricians and Gynecologists of Canada.^[17]^ Patients combined with severe cardiac or hepatic or renal diseases will not be taken into account in this study.

### 2.4 Types of interventions

Those RCTs taking acupoint catgut embedding therapy as the sole intervention or a major part of combination treatment with other interventions such as Chinese herbal medicine, western medicine, or other external therapies of TCM will be included.

### 2.5 Types of comparisons

The control group will include blank control, placebo control, and non-acupoint catgut embedding conventional therapies control, including Chinese herbal medicine, western medicine, and common acupuncture. If acupoint catgut embedding is combined with western medicine in the experimental group, the control group must use this western medicine in the same way.

### 2.6 Types of outcome measures

The primary outcomes include:

1. Visual analog scale (VAS): the measurement of pain before and after the treatment period.
2. Dysmenorrhea symptom score: the measurement of remission of dysmenorrhea symptoms before and after the treatment period.
3. Clinical effectiveness rate: an overall relief to menstruation-related symptoms measured by changes of relevant scale scores or self-report before and after the treatment period.

The secondary outcomes include:

1. Adverse events: fever, infection, bruising, numbness, local pain or ulcer, and so on.
2. Recurrence rate.

### 2.7 Literature search strategy

We will be comprehensively searched 4 international electronic databases (PubMed, Cochrane Library, EMBASE, and Web of Science) and 4 Chinese electronic databases (CNKI, VIP, Wanfang, and CBM) for relevant literature. We only include studies published from the initiation to September 2020. These studies must be published in English or Chinese. The literature search will be constructed around search terms for acupoint catgut embedding, PD and RCTs, and adapted for each database as necessary. The references to the included studies will also be screened for further material for inclusion. The detailed search strategy for PubMed is shown in Table 1. It will also be applied to other electronic databases.

**Table 1:** Search strategy used in PubMed database.

Serial number	Search items
#1	Dysmenorrhea
#2	Menstrual Pain^∗^
#3	Painful Menstruation^∗^
#4	Primary Dysmenorrhea
#5	Menstrual discord
#6	OR #1-#5
#7	Catgut Embedding
#8	Catgut Embedding Therapy
#9	Acupoint Catgut Embedding
#10	Catgut Implantation At acupuncture point
#11	Catgut Implantation At Acupoint
#12	Catgut Implantation
#13	Point Embedding Therapy
#14	Acupoint Thread-embedding
#15	OR #7-#14
#16	#6 AND #15

### 2.8 Data selection

**Figure 1:**
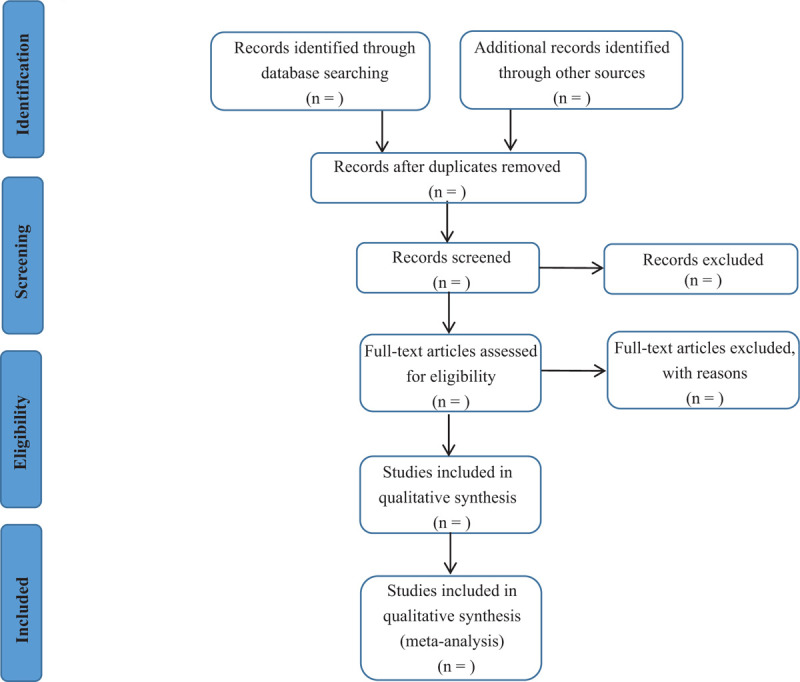
Flow diagram of study selection process.

In the data selection, 2 team members (Xue Wang and Nan Wang) will independently operate all literature search strategies. In accordance with the inclusion criteria and exclusion criteria, all eligible studies titles and abstracts will be screened for relevance while all clearly irrelevant ones will be discarded. If the result is not clearly irrelevant, the full text will be downloaded. Any differences between the reviewers on whether or not to include a specific study would be settled by a discussion with a third reviewer (Junquan Liang). Data extraction items include information about the first author, publication time, sample size, participant demographics and baseline characteristics, randomization, blinding, intervention details, control details, treatment period, outcome, adverse events, follow-up period, and recurrence rate. Lacking information will be requested by contacting the writer of the original article. The flow diagram of the study selection process is displayed in Figure 1.

### 2.9 Assessment of risk of bias

The quality of the included studies will be evaluated with the Cochrane Handbook 5.1.0 bias risk assessment tool. There are 5 aspects of bias evaluation: ① selection bias (random sequence generation or allocation concealment), ② performance bias and detection bias (blinding of participants, personnel, and outcome assessment), ③ attrition bias (incomplete outcome data), ④ reporting bias (selective reporting), ⑤ other biases.^[16]^ Assessment results of the risk of bias will be represented as follows: L (low), U (unclear), and H (high). Differences will be settled by discussion among all authors.

### 2.10 Data synthesis

Review Manager 5.3 software would be used to conduct the quantitative synthesis if included studies are sufficiently homogeneous. Given a 95% confidence interval (CI), mean difference (MD) or standardized mean difference (SMD) will be used for continuous data, while risk ratio (RR) will be applied to the analysis of dichotomous data. In the case of homogeneous data, if *I*^2^ ≤ 50%, *P* > .1, the fixed-effect model will be employed for the meta-analysis. If not, we will further analyze the sources of heterogeneity. After ruling out the impact on apparent heterogeneity, a random effect model will be employed to conduct the meta-analysis. Analyses of sensitivity and bias risk will also be carried out. A general descriptive analysis of adverse reactions will be made at last.

#### 2.10.1 Analysis of subgroups

There are some planned subgroup analyses that will be performed: different kinds of treatment methods (Chinese herbal medicine, acupuncture and moxibustion combined with Chinese herbal medicine, acupoint catgut embedding), different treatment periods and different follow-up periods (≤3 months, 3–6 months, >6 months).

#### 2.10.2 Sensitivity analysis

Sensitivity analysis will be carried out to determine the robustness and stability of pooled results by ruling out the studies of lower quality.

#### 2.10.3 Reporting bias analysis

If there are 10 or more trials in the analysis, reporting bias would be assessed by a funnel plot. When asymmetry shows in the visual examination, the Egger method could be carried out for exploratory analysis.

#### 2.10.4 Quality of evidence

We will assess the quality of evidence for the outcomes by Grading of Recommendations Assessment, Development, and Evaluation (GRADE) system. These 5 items (limitations, inconsistency, indirectness, imprecision, and publication bias) will be used to evaluate each outcome.^[18]^

### 2.11 Ethics and dissemination

There is no ethical approval requirement for this study because of no need for individual patients date in our study. Besides, the findings of this systematic review will be published in a peer-reviewed journal.

## 3 Discussion

It is reported that dysmenorrhea affects nearly 90% of childbearing-age women in various degrees. Sometimes it could severely affect patients living quality and work efficiency, leading to economic loss and social burden. Due to dysmenorrhea, the annual economic loss is estimated at approximately 600 million work hours and worth $2 billion in the US. It has widespread impacts more than the socio-economic one.^[10,19]^ But along with many adverse reactions, the conventional western medicines do not have a good long-term effect. Therefore, it is important to find a treatment which is effective and has fewer side effects for PD. In recent years, a growing number of PD patients are seeking help from alternative treatment.

In TCM, insufficiency or obstructed qi and blood of the thoroughfare vessel and conception vessel are considered as the primary cause of PD.^[6]^ Studies have signified that acupuncture can relieve pain by activating qi and blood circulation, and is effective for PD treatment. As a modern part of acupuncture, acupoint catgut embedding therapy has the advantages of convenient operation, long treatment interval, and reduced medical frequency, which is more suitable for modern womens irregular and busy life. And it has been shown that acupoint catgut embedding can effectively reduce prostaglandin level, which has a close relationship with the occurrence of PD.^[12]^

Currently, acupoint catgut embedding therapy has been extensively used in the treatment of PD. More and more relevant studies are carrying out worldwide. The latest meta-analysis of this was published 4 years ago.^[14]^ However, the inferior quality of evidence and publication only in Chinese were its limitations. A high-quality English review and meta-analysis of acupoint catgut embedding treating PD has not appeared. In the past 4 years, there have been some new reports on the RCTs of acupoint catgut embedding in treating PD. Many previous relevant RCTs with low quality can be replaced by new ones for a newer and better review and analysis. Thus, we believe it is necessary to conduct the systematic review and meta-analysis. The purpose of this study is to identify available RCTs of acupoint catgut embedding therapy on PD to offer persuasive evidence of effectiveness and safety for clinical practitioners, scientific researchers, and even general patients.

## Acknowledgments

The authors thank the National Natural Science Foundation of China (grant No. 81473755, Guizhen Chen; 81574064, Yunxiang Xu), the Shenzhen Science and Technology Planning Project (grant No. JCYJ20170306152650625), and the research funding of Affiliated Bao’an Hospital of Guangzhou University.

## Author contributions

**Conceptualization:** Xue Wang, Junquan Liang, Yunxiang Xu, Guizhen Chen.

**Data curation:** Xue Wang, Nan Wang, Junquan Liang, Yunxiang Xu.

**Formal analysis:** Xue Wang, Nan Wang.

**Investigation:** Xue Wang, Nan Wang.

**Methodology:** Xue Wang, Nan Wang, Junquan Liang.

**Project administration:** Yunxiang Xu, Guizhen Chen.

**Resources:** Xue Wang, Nan Wang, Junquan Liang.

**Software:** Xue Wang, Nan Wang.

**Supervision:** Yunxiang Xu, Guizhen Chen.

**Visualization:** Xue Wang, Nan Wang.

**Writing – original draft:** Xue Wang, Nan Wang, Junquan Liang.

**Writing – review & editing:** Xue Wang, Yunxiang Xu, Guizhen Chen.
